# Secondary alveolar bone grafting using autologous versus alloplastic material in the treatment of cleft lip and palate patients: systematic review and meta-analysis

**DOI:** 10.1186/s40510-018-0252-y

**Published:** 2019-02-11

**Authors:** A. Scalzone, C. Flores-Mir, D. Carozza, F. d’Apuzzo, V. Grassia, L. Perillo

**Affiliations:** 1 Orthodontic Division, Multidisciplinary Department of Medical-Surgical and Dental Specialties, University of Campania “Luigi Vanvitelli”, Via Luigi De Crecchio 6, 80138 Naples, Italy; 2 Department of Dentistry, University of Alberta, Edmonton, Canada

**Keywords:** Bone grafting, Nonsyndromic clefting, Unilateral cleft lip and palate, Computerized tomography

## Abstract

**Background:**

A systematic review assessing autologous versus alloplastic bone for secondary alveolar bone grafting in patients with cleft lip and palate was published in 2011 and included only one randomized controlled trial comparing traditional iliac bone graft to recombinant human bone morphogenetic protein-2 (rh-BMP2).

**Objectives:**

To perform a systematic review with meta-analysis on the use of secondary alveolar bone grafting (autologous bone and rh-BMP2 graft) in order to improve bone volume and height in patients with cleft lip and palate.

**Data sources:**

An electronic search was conducted via PubMed/MEDLINE, Cochrane Central Register of Controlled Trials (CONTROL) via Cochrane Library, EMBASE via Ovid, and LILAC for studies published between January 2008 and September 2018. The systematic review registration number at PROSPERO was 42018085858.

**Eligibility criteria:**

Only RCTs were included. Inclusion criteria were patients with the diagnosis of unilateral cleft lip and palate older than 5 years of age, radiographic evaluation (CT and/or CBCT) of the cleft area, and at least a 6-month follow-up.

**Main outcome measures:**

Bone formation and bone height by radiographic CT evaluation (preoperatively, after 6 months and after 1 year of follow-up) and length of hospital stay were assessed.

**Results:**

Four studies met strict inclusion criteria. Autologous bone graft showed statistically significant higher bone formation after 6-month follow-up (MD − 14.410; 95% CI − 22.392 to − 6.428; *p* = 0.000). No statistically significant difference was noted after a 1-year follow-up (MD 6.227; 95% CI − 15.967 to 28.422; *p* = 0.582). No statistically significant difference in bone height was noted after 6-month (MD − 18.737; 95% CI − 43.560 to 6.087; *p* = 0.139) and 1-year follow-up (MD − 4.401; 95% CI − 30.636 to 21.834; *p* = 0.742). Patients who underwent rh-BMP2 graft had a statistically significant reduced hospital stay (MD − 1.146; 95% CI − 2.147 to − 0.145; *p* = 0.025).

**Limitations:**

The main limitation is the high risk of bias among included studies.

**Conclusion:**

Autologous bone and rh-BMP2 graft showed a similar effectiveness in maxillary alveolar reconstruction in patients with unilateral cleft lip and palate assessing bone graft volume and height although rh-BMP2 graft showed a relative shorter length of hospital stay (high uncertainty level).

**Electronic supplementary material:**

The online version of this article (10.1186/s40510-018-0252-y) contains supplementary material, which is available to authorized users.

## Introduction

### Rationale

Secondary alveolar bone grafting (SABG) remains one of the main challenges during interdisciplinary orthodontic management in cases with bone defects [1]. Nevertheless, it is a well-established procedure for the management of patients with alveolar cleft [2, 3]. In these cases, alveolar repair is performed typically during mixed dentition phase (between 7 and 12 years old) before permanent canine eruption [4–6], whereas the timing of graft placement is based more on dental development than chronological age [7, 8]. Some of the main advantages of performing a bone graft are to provide osseous support for the teeth near the area of the cleft facilitating eruption of the teeth and to fuse the segments of the maxillary arch and alveolar ridge, enhancing lip support and improving facial esthetics [9–12].

Many sources of bone both autologous and alloplastic have been studied and compared. The question of the preferred donor site for cleft grafts has been debated for many years [13]. Autologous bone graft has always been the gold standard of bone replacement because it provides osteogenic cells as well as essential factors needed for bone healing and regeneration. It can be taken from the patient’s iliac crest, mandible, or tibia. The choice between these sites is influenced by several factors including the surgeon’s experience, the volume of bone required, and the morbidity of the harvest area [14]. To avoid or reduce such morbidity, surgeons are searching for a bone graft substitute. In 2007, the recombinant human bone morphogenetic protein-2 (rhBMP-2) started to be used after the Food and Drug Administration approval as an alternative to autologous bone graft for localized alveolar ridge augmentation [15]. The identification and development of rhBMP-2 associated with a collagen sponge carrier has allowed the use of alloplastic bone graft. The bone morphogenetic proteins (BMPs) play a role in osteogenesis and chondrogenesis and are also involved in embryonic development and fracture healing [16]. It is important to better understand if rhBMP-2 graft could represent an autologous bone graft substitute, thus eliminating donor site morbidity [17]. The last systematic review assessing autologous vs. alloplastic bone for SABG in patients with cleft lip and palate was published by Guo et al. in 2011 [18] that included only one randomized controlled trial (RCT) comparing traditional iliac bone graft to rhBMP-2. Therefore, the objectives of this systematic review are to evaluate volume and bone height and length of hospital stay in patients with unilateral cleft lip and palate treated with autologous or rhBMP-2 secondary bone graft, thus synthesizing the available evidence on the effectiveness of the two types of treatment.

## Methods

This systematic review followed the PRISMA Guidelines checklist (Additional file 1) [19]. The protocol was registered at PROSPERO, the international prospective register of systematic reviews (Centre for Reviews and Dissemination, University of York, UK) under the number 42018085858. This study was approved by the Ethics Committee of the University of Campania “Luigi Vanvitelli” (Prot. n. 21808/17).

### Eligibility criteria

#### Types of studies

Only RCTs were included.

#### Types of participants

Inclusion criteria were patients with the diagnosis of unilateral cleft lip and palate older than 5 years of age, radiographic evaluation (CT and/or CBCT) of the cleft area, and at least a 6-month follow-up. Exclusion criteria were edentulous maxilla, atypical or non-described cleft diagnosis, and associated syndrome conditions.

### Types of outcomes

Primary outcomes were the radiographic assessment of bone graft volume through the 3D images and the radiographic assessment in the grafted area of alveolar bone height through the 3D images.

The secondary outcome was the length of hospital stay.

### Search methodology

There was no language restriction set. An electronic search was independently conducted by two authors (AS and DC) via PubMed/MEDLINE, using the Medical Subject Headings (MeSH) terms “secondary alveolar bone graft,” “alveolar bone graft,” “cleft lip,” “cleft palate,” “cleft lip and palate,” “alveolar bone graft cleft,” “autologous bone graft cleft,” “alloplastic bone graft cleft,” “BMP-2 graft cleft,” and “bone graft cleft” for studies published between January 2008 and September 2018. A similar search was conducted on Cochrane Central Register of Controlled Trials (CONTROL) via Cochrane Library, EMBASE via Ovid, and LILAC.

A secondary search was conducted by reading the reference lists of the articles meeting the inclusion criteria for eventual additional studies relevant to this review. The full-text copies were then independently assessed by the two reviewers, and any disagreement on the eligibility of included studies was resolved through consensus. Studies that did not match the inclusion criteria in this second selection phase were excluded.

### Study selection

The titles and abstracts of all studies resulting from the search were independently and randomly assessed by the two reviewers (AS and DC) by scanning the titles, abstracts, and the keywords of the studies in the search results. Full copies of all apparently relevant studies or those for which there were insufficient data in the title and abstract to make a clear decision were obtained. The full-text copies were then independently assessed by two reviewers, and any disagreement on the eligibility of included studies was resolved through consensus. Studies that did not match the inclusion criteria in this second selection phase were excluded (Table 1) [11, 20–23].

**Table 1:** Characteristics of excluded studies

Study	Reason for exclusion
Alonso 2014	No information about bone healing, volume, or morbidity
Ganesh 2015	No information about bone healing, volume, or morbidity
Ayoub 2016	No control group
Chang 2016	No control group
Raposo-Amaral 2016	No information about bone healing, volume, or morbidity

### Data collection process

The two reviewers independently extracted data from the included studies. Data extracted were details of the study setting, characteristics of the study samples, graft sources, and outcomes. If stated, the sources of funding of any included studies were recorded. Extracted data from the included studies is presented in Table 2.

**Table 2:** Characteristics of included studies

Study	Setting	Total sample recruited	Mean age (years)	Study groups (*n*)	Outcomes	Reference
Dickinson 2008	University of California, Los Angeles Medical Center and Olive view Medical Center	21: male 9, female 12	16.1	Intervention group: the InFuse® bone graft (Sofamor-Danek, Memphis, Tennessee), which is a collagen matrix impregnated with rhBMP-2, were grafted (9) Control group: traditional iliac crest graft (12)	Alveolar ridge healing (preoperative, 6 weeks and 1 year after surgery); three-dimensional computed tomographic scan results (preoperative and 1 year after surgery); volume of bone filled in alveolar cleft (preoperative, 6 weeks and 1 year after surgery); length of hospital stay; donor site pain intensity and frequency (VAS) (day 1, day 7, week 3, week 6, month 3; cost of surgery)	[25]
Alonso 2010	Craniofacial Surgery Unit of the University of Sao Paulo Medical School and CAIF (Assistance Center for Cleft Lip and Palate)	16: male 9, female 7	9.6	Intervention group: InFuse® bone graft (Medtronic, Memphis, Tennessee), a collagen sponge carrier with lyophilized rhBMP-2 (8) Control group: traditional iliac crest graft (8)	Three-dimensional computed tomographic scan results (preoperative, 6 months and 12 months after surgery); volume of bone filled in alveolar cleft (preoperative, 6 weeks and 1 year after surgery); donor site pain intensity and frequency (VAS) (day 1, day 7, week 3, week 6, month 3)	[26]
Canan 2012	University of Sao Paulo Medical School, Brazil	18: male 12, female 6	From 8 to 15	Intervention group: the InFuse® bone graft (Medtronic, Memphis, Tennessee), which is an absorbable collagen sponge with rhBMP-2, were grafted (6) Control group: traditional iliac crest graft (6) Periosteoplasty group: (6)	Three-dimensional computed tomographic scan results (preoperative, 3 months, 6 months, and 12 months after surgery); volume of bone filled in alveolar cleft (preoperative, 3 months, 6 months and 12 months after surgery); maxillary height repair (preoperative, 3 months, 6 months, and 12 months after surgery)	[4]
Neovius 2013	Karolinska University Hospital, Stockholm Craniofacial Centre, Sweden	7: male 6, female 1	9.11	Intervention group: rhBMP-2 delivered by an hydrogel carrier was grafted (4) Control group: traditional iliac crest graft (3)	Three-dimensional computed tomographic scan results (preoperative, 6 months after surgery); volume of bone filled in alveolar cleft (preoperative, 6 months after surgery); length of hospital stay; donor site morbidity; surgery time	[27]

### Risk of bias in individual studies

**Fig. 1:**
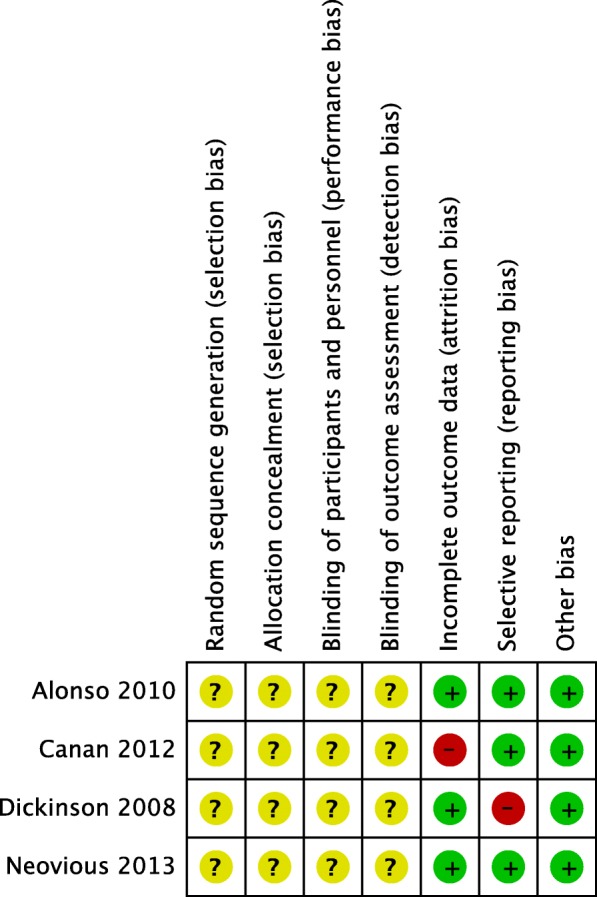
Risk of bias summary: risk of bias item for each included study according to the Cochrane Collaboration Tool

Assessment of risk of bias in included studies was undertaken independently by two reviewers. The Cochrane Collaboration’s tool for assessing risk of bias was used (Fig. 1). Disagreements in the classification were resolved by discussion. The following domains were assessed as “low,” “high,” or “unclear” risk of bias: random sequence generation, allocation concealment, blinding of participants and personnel, blinding of outcome assessment, incomplete outcome data, selective reporting, and other biases.

### Summary measures

For descriptive continuous data (bone volume, bone height, length of hospital stay), mean, standard deviation (SD), sample size, and weighted mean differences are reported. A *p* value of less than 0.05 was considered statistically significant. Random effects meta-analysis was performed using Comprehensive Meta-Analysis V3 software (Biostat, USA). Statistical heterogeneity was calculated by inconsistency indexes (I2), and a value greater than 50% will be considered an indicator of substantial heterogeneity between studies. The significance level was set at 5.0%.

### Additional analysis

Subgroup analysis was conducted to explore the influence of the characteristics in the groups of studies, such as patients’ age, on the meta-analysis outcomes, thus to produce estimates and formal statistical comparisons across the subgroups.

### Risk of bias across studies

The grading of recommendation, assessment, development, and evaluation (GRADE) instrument assessed evidence quality and grading of recommendation strength in the included studies in the quantitative synthesis and meta-analysis. This assessment was based on considerations such as study design, consistency, directness, precision, publication bias, and other aspects reported by studies included in this systematic review. The quality of the evidence was characterized as high, moderate, low, or very low and was assessed using tools from the website http://gradepro.org [24].

## Results

### Study selection

**Fig. 2:**
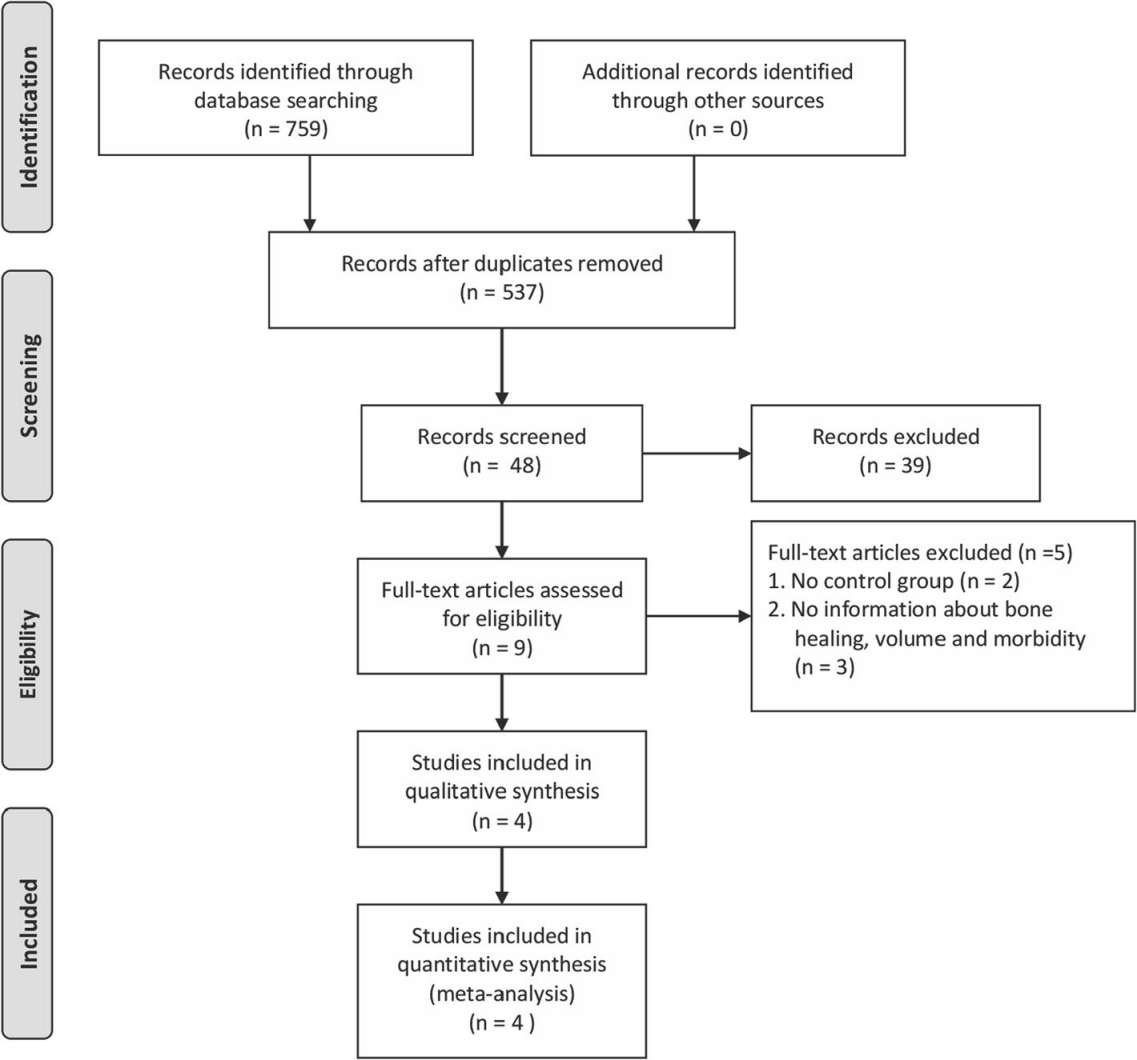
Flow diagram of study inclusion

After the search strategies, 759 publications were identified, of which 750 were excluded after reviewing the titles and abstracts. Of the remaining 9 publications, full texts were obtained. After screening full texts, 5 studies were excluded. Therefore, only 4 RCTs [4, 25–27] fulfilled all the inclusion criteria. For details of the studies examined and reasons for inclusion and exclusion, please see Tables 1 and 2. The process of study identification is presented in Fig. 2.

### Study characteristics

A total of 56 patients with unilateral cleft lip and palate was involved, 27 underwent rhBMP-2 graft, while 29 patients underwent iliac crest bone graft. From the 4 selected studies, 2 were conducted in Brazil, 1 in the USA, and 1 in Sweden. In 3 of these studies, the intervention group received the rhBMP-2 delivered by a collagen sponge carrier and the control group received traditional iliac crest cancellous graft. In only 1 study, the intervention group received the rhBMP-2 delivered by a hydrogel carrier.

Two of the studies are assessed as being mainly at “unclear” and the other two at “high” risk of bias. Details of the risk of bias assessment are noted below:

1. *Random sequence generation*: All the studies mentioned random allocation, but none mentioned the detail of sequence generation. Thus, the sequence generation was not clear.
2. *Allocation concealment*: None of the included studies clearly described any allocation concealment.
3. *Blinding of participants and personnel*: None of the studies mentioned whether the surgeon or participants were blinded, so blinding was also considered to be unknown.
4. *Blinding of outcome assessment*: None of the studies mentioned blinding of outcome assessors.
5. *Incomplete outcome data*: From all the studies, there were no reported dropouts. The only not reported data were the preoperative defect sizes of cleft lip and palate in Canan et al. [4].
6. *Selective reporting*: In Dickinson et al. [25], some of the variables mentioned in “materials and methods” were not fully reported in “results.”
7. *Other biases*: We did not find any other source of bias.

### Synthesis of results

The included studies compared traditional autologous bone graft (iliac crest) with alloplastic bone graft (rhBMP-2). They evaluated bone formation and bone height by CT evaluation and length of hospital stay after surgery. Three-dimensional CT evaluations were obtained preoperatively, after 6 months and after 1-year follow-up.

#### Bone graft volume

**Fig. 3:**
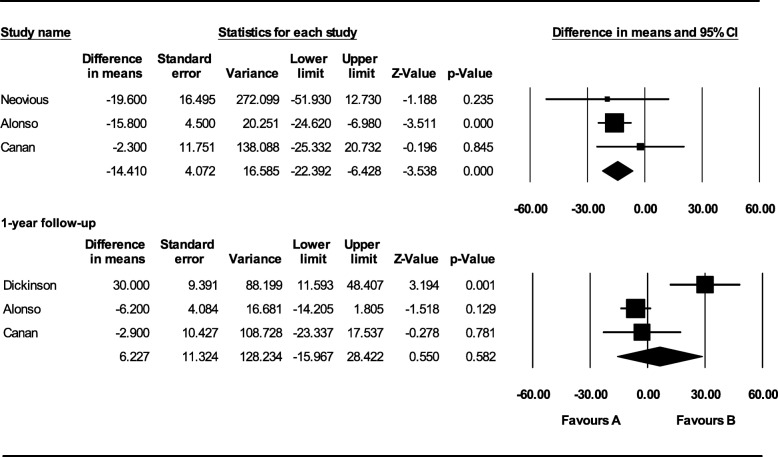
Comparison of radiographic assessment between autologous bone and rh-BMP2 graft: bone graft volume after 6-month and 1-year follow-up [favors A: autologous bone graft; favors B: alloplastic bone graft]

Neovius et al., Alonso et al., and Canan et al. assessed bone graft volume with CT evaluation after 6 months. Autologous bone graft showed higher bone formation after a 6-month follow-up period that resulted to be statistically significant (MD − 14.410; 95% CI − 22.392 to − 6.428; *p* = 0.000) (Fig. 3).

Alonso et al., Canan et al., and Dickinson et al. assessed bone graft volume with CT evaluation after 1 year. No statistically significant difference was noted (MD 6.227; 95% CI − 15.967 to 28.422; *p* = 0.582) (Fig. 3).

**Fig. 4:**
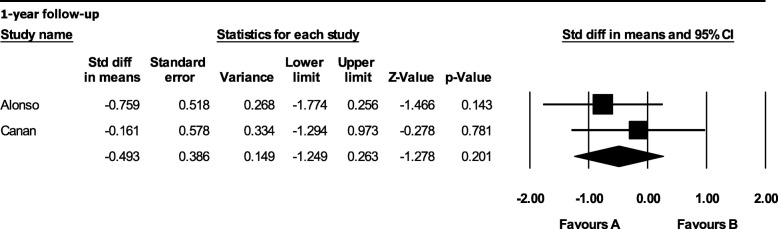
Subgroup analysis of radiographic assessment between autologous bone and rh-BMP2 graft: bone graft volume after a 1-year follow-up considering the patient’s age [favors A: autologous bone graft; favors B: alloplastic bone graft]

A subgroup analysis was also performed to evaluate the influence of patients’ mean age on the meta-analysis outcome. Dickinson’s sample had a higher mean age (16.1 years) compared to the other studies. They reported that bone formation with alloplastic bone graft was significantly higher than that with autologous bone graft after a 1-year follow-up (MD 30.000; 95% CI 11.593 to 48.407; *p* = 0.001). After performing a subgroup analysis without Dickinson’s data, still, no statistically significant difference was noted (MD − 0.493; 95% CI − 1.249 to 0.263; *p* = 0.201) (Fig. 4).

#### Bone graft height

**Fig. 5:**
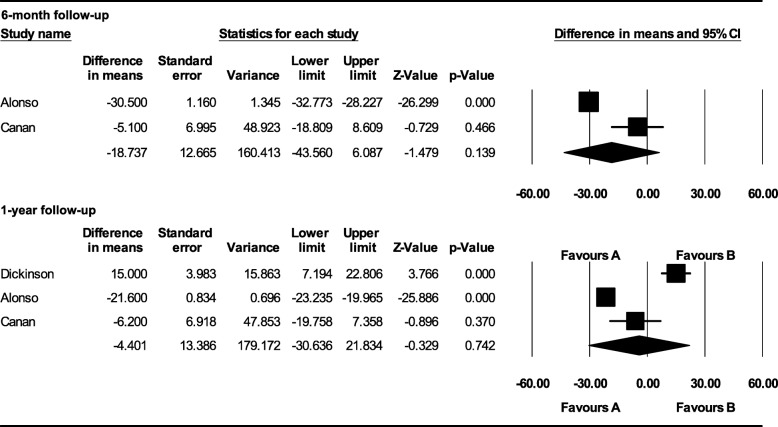
Comparison of radiographic assessment between autologous bone and rh-BMP2 graft: bone graft height after 6-month and 1-year follow-up [favors A: autologous bone graft; favors B: alloplastic bone graft]

**Fig. 6:**
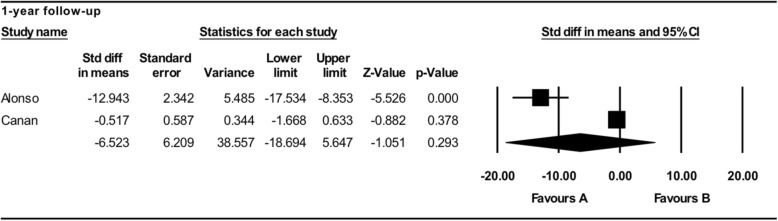
Subgroup analysis of radiographic assessment between autologous bone and rh-BMP2 graft: bone graft height after a 1-year follow-up considering the patient’s age [favors A: autologous bone graft; favors B: alloplastic bone graft]

Alonso et al. and Canan et al. assessed bone height with CT evaluation after 6 months. No statistically significant difference was noted after 6 months (MD − 18.737; 95% CI − 43.560 to 6.087; *p* = 0.139) (Fig. 5). Dickinson et al., Alonso et al., and Canan et al. assessed bone height with CT evaluation after 1 year. No statistically significant difference was noted (MD − 4.401; 95% CI − 30.636 to 21.834; *p* = 0.742) (Fig. 5). A subgroup analysis was also performed to evaluate the influence of patients’ mean age on the meta-analysis outcome. Dickinson’s sample had a higher mean age (16.1 years) compared to the other studies. In particular, considering the studies with a similar age range, the rhBMP-2 graft reported not statistically significant results in bone height than autologous bone graft after a 1-year follow-up (MD − 6.523; 95% CI − 18.694 to 5.647; *p* = 0.293) (Fig. 6).

#### Length of hospital stay

**Fig. 7:**
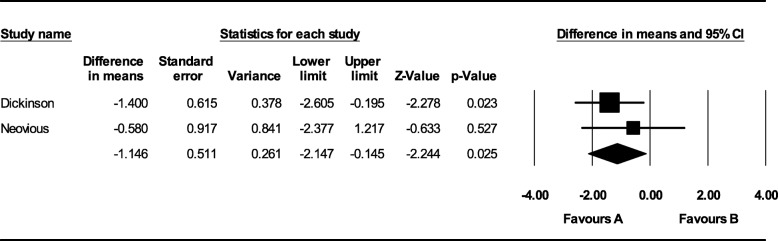
Comparison of length of hospital stay between autologous bone and rh-BMP2 graft [favors A: autologous bone graft; favors B: alloplastic bone graft]

Dickinson et al. and Neovius et al. assessed the mean length of hospital stay after surgery. Patients who underwent rhBMP-2 graft had a reduced hospital stay, and the difference of about 1 day resulted as statistically significant (MD − 1.146; 95% CI − 2.147 to − 0.145; *p* = 0.025) (Fig. 7).

### Risk of bias across studies

Overall, the quality of the evidence from the outcomes evaluated by the GRADE system was assessed as low, suggesting moderate confidence in the estimated effect from the assessed outcomes.

An “unclear” or “high” risk of bias among the included studies was the main factor responsible for the limited quality of the evidence (Fig. 1). Publication bias was not assessed due to the limited number of studies included.

## Discussion

### Summary of evidence

This systematic review included four RCTs [4, 25–27] that focused on the effectiveness of autologous and alloplastic SABG in children with cleft lip and palate after a 6-month and/or 1-year follow-up. Each included RCT used iliac bone as autologous donor site material and rhBMP-2 as alloplastic material. The three-dimensional radiographic evaluation with CT is the only reliable and elected method for the analysis of height and volume of the alveolar bone [4, 28]. The comparison of bone volume between autologous and alloplastic bone graft after a 6-month follow-up showed statistically significant results favoring the autologous approach over the rhBMP-2 graft, but after 1-year follow-up, the differences in bone formation disappeared. It has to be noted that the results favored the autologous bone graft when excluding Dickinson’s study [25], probably because it only considered patients after the eruption of the permanent canine, whereas the other studies involved patients before canine eruption. Another possible explanation is that there seems to be a tendency of autologous bone graft to lose its volume from 6 months on, most probably owing to bone resorption until the canine erupts [4]. The assessment of bone graft height after 6-month and 1-year follow-up showed no statistically significant results between autologous versus rhBMP-2 bone graft at neither time frame.

Regarding the length of hospital stay, the patients treated with autologous bone graft were hospitalized longer than patients undergoing an rhBMP-2 graft with a statistical significance. This is likely related to wound healing from the harvested bone from the iliac crest region. However, the difference of hospital stay was about 1 day; thus, it should not be considered of substantial clinical significance. It has been recommended that it may be important to find an autologous bone graft substitute, though partial, to avoid the need for a second surgical site reducing operation time, hospital stay, and postoperative pain with any related complication, thus making the procedure more acceptable for the patient [16]. The current synthesis does not favor either approach at 1-year follow-up. The only difference is an additional day of hospital stay. No data assessing differences after more than 1 year were identified. According to the GRADE working group, the quality of the evidence in this research was graded as moderate (Additional files 2 and 3). A systematic review qualified as moderate indicated that the readers should not be completely confident that the results of this meta-analysis necessarily reflect every day responses. The review conclusions should be interpreted with caution, due to the limited number of studies included and some risk of different types of bias. Above all, the sample size of the RCTs included was small, in particular in Neovius’ study that only included seven patients in total. Thus, further well-conducted multicentric prospective controlled randomized clinical studies with larger sample size, similar treatment conditions, and continued follow-up of the two methods are needed in order to recommend the proper bone graft technique in patients with cleft lip and palate in an evidence-based and predictable way.

The main limitation of our meta-analysis is the lack of adequate methodological quality among the included studies. First, each study did not describe the sequence generation, although they claimed that the groups in their trails were all randomly allocated and this might have introduced some selection bias. Second, the included studies did not mention the allocation concealment method, and neither of them clearly stated how blinding was used during the trial. Moreover, in these RCTs, the surgical procedures used for the bone graft management in each different study center were not standardized. This may have affected the outcomes, causing a risk of performance bias. Finally, all the RCTs included in the systematic review utilized the Infuse® Bone Graft with a collagen sponge with lyophilized rhBMP-2, except Neovius et al. that performed the alloplastic bone graft using rhBMP-2 with an hydrogel carrier. Hence, the results cannot be extrapolated to other alloplastic materials or autologous bone harvest areas.

## Conclusion

The absence of significant differences between autologous bone graft and rhBMP-2 graft assessing bone graft volume and height showed a similar effectiveness of the methods in maxillary alveolar reconstruction in patients with unilateral cleft lip and palate. An additional day of hospital stay for the patients was noted when performing an autologous bone graft. However, the difference may not be considered clinically relevant (moderate level of evidence).

## Additional files

Additional file 1: PRISMA 2009 Checklist. (PDF 68 kb)

Additional file 2: GRADE evidence profile. (PDF 319 kb)

Additional file 3: Summary of findings. (PDF 773 kb)

## Abbreviations

CBCT: Cone beam computerized tomography
CI: Confidence interval
CT: Computerized tomography
GRADE: Grading of recommendation, assessment, development, and evaluation
MD: Mean difference
PRISMA: Preferred reporting items for systematic reviews and meta-analysis
PROSPERO: Prospective register of systematic reviews protocols
RCT: Randomized controlled trial
rh-BMP2: Recombinant human bone morphogenetic protein-2
SABG: Secondary alveolar bone grafting
SD: Standard deviation

## References

[1] Boyne PJ, Sands NR (1972). Secondary bone grafting of residual alveolar and palatal clefts. J Oral Surg.

[2] Mikoya T, Inoue N, Matsuzawa Y, Totsuka Y, Kajii TS, Hirosawa T (2010). Monocortical mandibular bone grafting for reconstruction of alveolar cleft. Cleft Palate Craniofac J.

[3] Grassia V, Lombardi A, Kawasaki H, Ferri C, Perillo L, Mosca L, Delle Cave D, Nucci L, Porcelli M, Caraglia M (2018). Salivary microRNAs as new molecular markers in cleft lip and palate: a new frontier in molecular medicine. Oncotarget.

[4] Canan LW, da Silva FR, Alonso N, Tanikawa DYS, Rocha DL, Coelho JCU (2012). Human bone morphogenetic protein-2 use for maxillary reconstruction in cleft lip and palate patients. J Craniofac Surg.

[5] Mahajan R, Ghildiyal H, Khasgiwala A, Muthukrishnan G, Kahlon S (2017). Evaluation of secondary and late secondary alveolar bone grafting on 66 unilateral cleft lip and palate patients. Plast Surg (Oakv).

[6] Perillo L, Vitale M, d’apuzzo F, Isola G, Nucera R, Matarese G (2018). Interdisciplinary approach for a patient with unilateral cleft lip and palate. Am J Orthod Dentofac Orthop.

[7] Garcia MA, Yatabe M, Fuzer TU, Calvo AM, Trindade-Suedam IK (2018). Ideal versus late secondary alveolar bone graft surgery: a bone-thickness cone-beam computed tomographic assessment. Cleft Palate Craniofac J.

[8] Jeyaraj P, Sahoo NK, Chakranarayan A (2014). Mid versus late secondary alveolar bone grafting using iliac crest corticocancellous bone graft. J Maxillofac Oral Surg.

[9] Generali C, Primozic J, Richmond S, Bizzarro M, Flores-Mir C, Ovsenik M, Perillo L (2017). Three-dimensional evaluation of the maxillary arch and palate in unilateral cleft lip and palate subjects using digital dental casts. Eur J Orthod.

[10] Cho-Lee GY, Garcìa-Dìez EM, Rivera-Barò A (2013). Review of secondary alveolar cleft repair. Ann Maxillofac Surg.

[11] Raposo-Amaral CE, Denadai R, Alonso N (2016). Three-dimensional upper lip and nostril sill changes after cleft alveolus reconstruction using autologous bone grafting versus recombinant human bone morphogenetic protein-2. J Craniofac Surg.

[12] Vura N, Reddy KR, Sudhir R, Rajasekhar G, Kaluvala VR (2013). Donor site evaluation: anterior iliac crest following secondary alveolar bone grafting. J Clin Diagn Res.

[13] Murthy AS, Lehman JA (2006). Secondary alveolar bone grafting: an outcome analysis. Can J Plast Surg.

[14] Rawashdeh MA, Telfah H (2008). Secondary alveolar bone grafting: the dilemma of donor site selection and morbidity. Br J Oral Maxillofac Surg.

[15] McKay WF, Peckham SM, Badura JM (2007). A comprehensive clinical review of recombinant human bone morphogenetic protein-2 (Infuse Bone Graft). Inter Ortop.

[16] Van Hout WMMT, Van der Molen ABM, Breugem CC, Koole R, Van Cann (2011). Reconstruction of the alveolar cleft: can growth factor-aided tissue engineering replace autologous bone grafting? A literature review and systematic review of results obtained with bone morphogenetic protein-2. Clin Oral Investig.

[17] Allareddy V (2014). Use of human recombinant bone morphogenetic protein is associated with increased hospital charges in children with cleft lip and palate having bone graft procedures. J Oral Maxillofac Surg.

[18] Guo J, Li Li C, Zhang Q, Wu G, Deacon SA, Chen J, Hu H, Zou S, Ye Q (2011). Secondary bone grafting for alveolar cleft in children with cleft lip or cleft lip and palate. Cochrane Database Syst Rev.

[19] Moher D, Shamseer L, Clarke M, Ghersi D, Liberati A, Petticrew M, Shekelle P, Stewart LA, PRISMA-P Group (2015). Preferred reporting items for systematic review and meta-analysis protocols (PRISMA-P) 2015 statement. Syst Rev.

[20] Alonso N, Risso GH, Denadai R, Raposo-Amaral CE. Effect of maxillary alveolar reconstruction on nasal symmetry of cleft lip and palate patients: a study comparing iliac crest bone graft and recombinant human bone morphogenetic protein-2. J Plast Reconstr Aesthet Surg. 2014;67(9):1201-8. 10.1016/j.bjps.2014.05.014 24909628

[21] Ganesh P, Murthy J, Ulaghanathan N, Savitha VH. A randomized controlled trial comparing two techniques for unilateral cleft lip and palate: Growth and speech outcomes during mixed dentition. J Craniomaxillofac Surg. 2015;43(6):790-5. 10.1016/j.jcms.2015.03.033 25958096

[22] Ayoub A, Roshan CP, Gillgrass T, Naudi K, Ray A. The clinical application of rhBMP-7 for the reconstruction of alveolar cleft. J Plast Reconstr Aesthet Surg. 2016;69(1):101-7. 10.1016/j.bjps.2015.09.004 26507862

[23] Chang CS, Wallace CG, Hsiao YC, Chiu YT, Pai BC, Chen IJ, Liao YF, Liou EJ, Chen PK, Chen JP, Noordhoff MS. Difference in the Surgical Outcome of Unilateral Cleft Lip and Palate Patients with and without Pre-Alveolar Bone Graft Orthodontic Treatment. Sci Rep. 2016 4;6:23597. 10.1038/srep23597 PMC4819291 27041697

[24] Balshem H, Helfand M, Schünemann HJ, Oxman AD, Kunz R, Brozek J (2011). GRADE guidelines: rating the quality of evidence. J Clin Epidemiol.

[25] Dickinson BP, Ashley RK, Wasson KL, O’Hara C, Gabbay J, Heller JB, Bradley JP (2008). Reduced morbidity and improved healing with bone morphogenetic protein-2 in older patients with alveolar cleft defects. Plast Reconstr Surg.

[26] Alonso N, Tanikawa DYS, Freitas Rda S, Canan L, Rocha DL, Ozawa TO (2010). Evaluation of maxillary alveolar reconstruction using a resorbable collagen sponge with recombinant human bone morphogenetic protein-2 in cleft lip and palate patients. Tissue Eng Part C Methods.

[27] Neovius E, Lemberger M, Docherty Skogh AC, Hilborn J, Engstrand T (2013). Alveolar bone healing accompanied by severe swelling in cleft children treated with bone morphogenetic protein-2 delivered by hydrogel. J Plast Reconstr Aesthet Surg.

[28] Oberoi S, Chigurupati R, Gill P, Hoffman WY, Vargervik K (2009). Volumetric assessment of secondary alveolar bone grafting using cone beam computed tomography. Cleft Palat Craniofac J.

