# Supplementary material for: Secondary alveolar bone grafting using autologous versus alloplastic material in the treatment of cleft lip and palate patients: systematic review and meta-analysis
Source: Prog Orthod. 2019 Feb 11;20:6. doi: 10.1186/s40510-018-0252-y (PMC6369233; doi:10.1186/s40510-018-0252-y)
Supplement: Supplementary file 2 — GRADE evidence profile. (PDF 319 kb) [file 40510_2018_252_MOESM2_ESM.pdf]

Additional file 2. GRADE evidence profile

Secondary alveolar bone grafting in patients with cleft lip and palate											
**Certainty assessment**							**Summary of findings**				
**№ of participants (studies) Follow-up**	**Risk of bias**	**Inconsistency**	**Indirectness**	**Imprecision**	**Publication bias**	**Overall certainty of evidence**	**Study event rates (%)**		**Relative effect (95% CI)**	**Anticipated absolute effects**	
							**With Iliac crest**	**With BMP-2**		**Risk with Iliac crest**	**Risk difference with BMP-2**
**Bone volume (follow up: 6 months)**											
35 (3 RCTs)	serious [a]	not serious	not serious	not serious	none	⨁⨁⨁◯ MODERATE	17	18	-	The mean bone volume ranged from **48-76** %	MD **14.41 % fewer** (22.39 fewer to 6.42 fewer)
**Bone volume (follow up: 12 months)**											
49 (3 RCTs)	serious [a]	not serious	not serious	not serious	none	⨁⨁⨁◯ MODERATE	26	23	-	The mean bone volume ranged from **66-80** %	MD **6.22 % more** (15.96 fewer to 28.42 more)
**Bone height (follow up: 6 months)**											
28 (2 RCTs)	serious [a]	not serious	not serious	not serious	none	⨁⨁⨁◯ MODERATE	14	14	-	The mean bone height ranged from **64-83** %	MD **18.73 % fewer** (43.56 fewer to 6.08 more)
**Bone height (follow up: 12 months)**											
49 (3 RCTs)	serious [a]	not serious	not serious	not serious	none	⨁⨁⨁◯ MODERATE	26	23	-	The mean bone height ranged from **64-86** %	MD **4.4 % fewer** (30.63 fewer to 21.83 more)
**Hospital stay**											

CI: Confidence interval; **MD:** Mean difference

Explanations

a. All the studies are assessed as being at high risk of bias. Random sequence generation: All the studies mentioned random allocation but none mentioned the detail of sequence generation. Thus, the sequence generation was not clear. Allocation concealment: None of the included studies had clearly described the allocation concealment. Blinding of participants and personnel: None of the studies mentioned whether the surgeon or participants were blinded, s o blinding was also considered to be unknown. Blinding of outcome assessment: None of the studies mentioned blinding of outcome assessors Incomplete outcome data: From all the studies, there were no reported drop outs Selective reporting: In Dickinson 2008 some of the variables mentioned in “materials and methods” were not fully reported in “results”. Other bias: We did not find any other source of bias.
