# Supplementary material for: Secondary alveolar bone grafting using autologous versus alloplastic material in the treatment of cleft lip and palate patients: systematic review and meta-analysis
Source: Prog Orthod. 2019 Feb 11;20:6. doi: 10.1186/s40510-018-0252-y (PMC6369233; doi:10.1186/s40510-018-0252-y)
Supplement: Supplementary file 3 — Summary of findings. (PDF 773 kb) [file 40510_2018_252_MOESM3_ESM.pdf]

Additional file 3. Summary of findings

Secondary alveolar bone grafting in patients with cleft lip and palate

Patient or population: Secondary alveolar bone graft
Setting:
Intervention: rhBMP2
Comparison: Iliac crest

Outcomes	**Anticipated absolute effects*** (95% CI)		Relative effect (95% CI)	N° of participants (studies)	Certainty of the evidence (GRADE)	Comments
	**Risk with Iliac crest**	**Risk with BMP-2**				
Bone volume follow up: 6 months	The mean bone volume ranged from **48-76** %	The mean bone volume in the intervention group was 14,41 % fewer (22,39 fewer to 6,42 fewer)	-	35 (3 RCTs)	⨁⨁⨁◯ MODERATE [a]	
Bone volume follow up: 12 months	The mean bone volume ranged from **66-80** %	The mean bone volume in the intervention group was 6,22 % more (15,96 fewer to 28,42 more)	-	49 (3 RCTs)	⨁⨁⨁◯ MODERATE [a]	
Bone height follow up: 6 months	The mean bone height ranged from **64-83** %	The mean bone height in the intervention group was 18,73 % fewer (43,56 fewer to 6,08 more)	-	28 (2 RCTs)	⨁⨁⨁◯ MODERATE [a]	
Bone height follow up: 12 months	The mean bone height ranged from **64-86** %	The mean bone height in the intervention group was 4,4 % fewer (30,63 fewer to 21,83 more)	-	49 (3 RCTs)	⨁⨁⨁◯ MODERATE [a]	
Hospital stay	The mean hospital stay ranged from **1.8-3.3** days	The mean hospital stay in the intervention group was 1,14 days fewer (2,14 fewer to 0,14 fewer)	-	26 (2 RCTs)	⨁⨁⨁◯ MODERATE [a]	

***The risk in the intervention group** (and its 95% confidence interval) is based on the assumed risk in the comparis on group and the **relative effect** of the intervention (and its 95% CI).

CI: Confidence interval; **MD:** Mean difference

GRADE Working Group grades of evidence
High certainty: We are very confident that the true effect lies close to that of the estimate of the effect
Moderate certainty: We are moderately confident in the effect estimate: The true effect is likely to be clos e to the estimate of the effect, but there is a possibility that it is substantially different
Low certainty: Our confidence in the effect estimate is limited: The true effect may be substantially different from the estimate of the effect
Very low certainty: We have very little confidence in the effect estimate: The true effect is likely to be substantially different from the estimate of effect

Explanations

a. All the studies are assessed as being at high risk of bias . Random sequence generation: All the studies mentioned random allocation but none mentioned the detail of sequence generation. Thus the sequence generation was not clear. Allocation concealment: None of the included studies had clearly described the allocation concealment. Blinding of participants and personnel: None of the studies mentioned whether the surgeon or participants were blinded, so blinding was also considered to be unknown. Blinding of outcome assessment: None of the studies mentioned blinding of outcome assessors Incomplete outcome data: From all the studies, there were no reported dropouts. Selective reporting: In Dickinson 2008 some of the variables mentioned in "materials and methods" was not fully reported in "results ". Other bias: We did not find any other source of bias.
